# Supplementary figures and images for: Evaluation of a connectivity-based imaging metric that reflects functional decline in Multiple Sclerosis
Source: PLoS One. 2021 Jun 8;16(6):e0251338. doi: 10.1371/journal.pone.0251338 (PMC8186801; doi:10.1371/journal.pone.0251338)

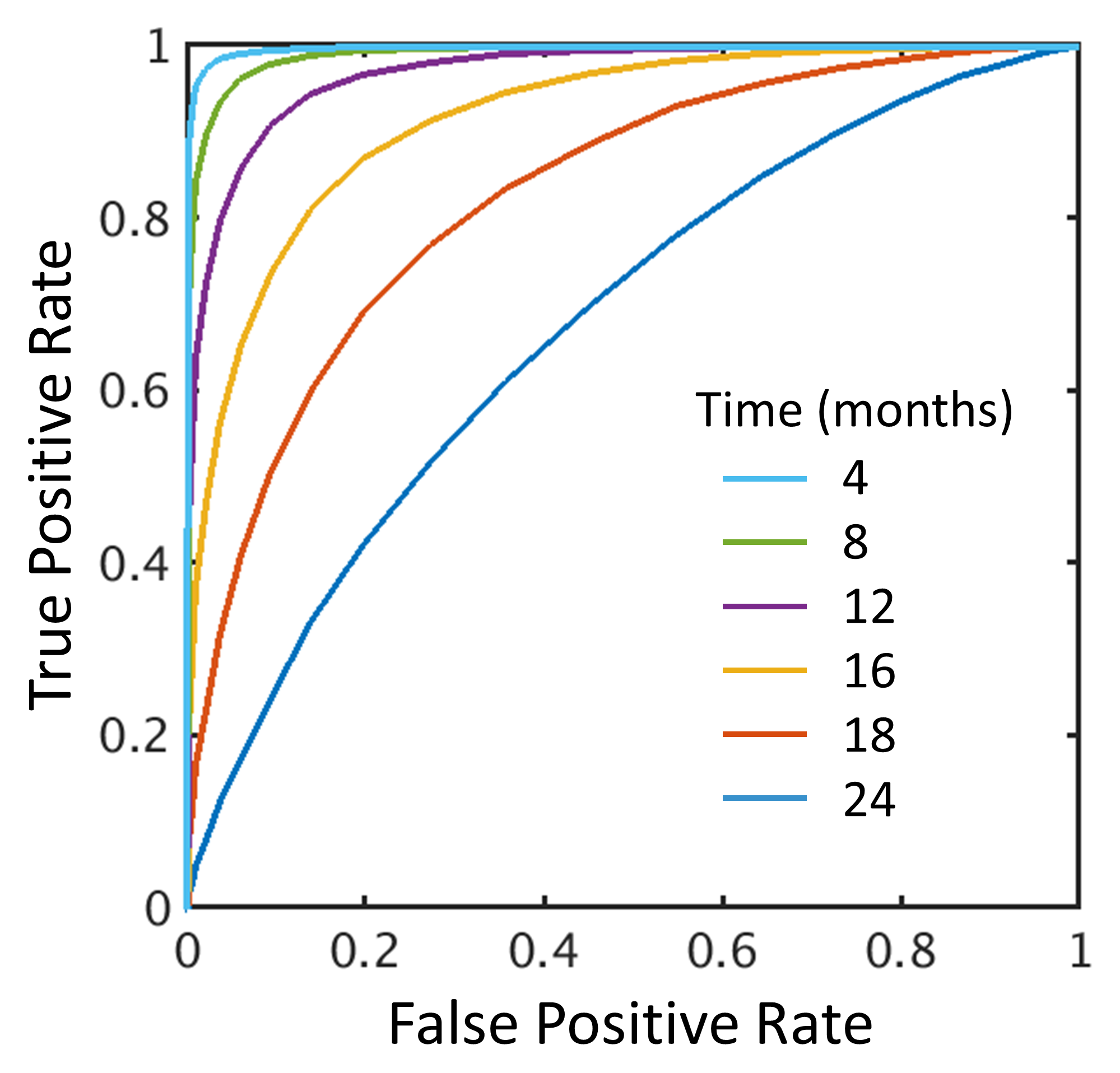

Supplement: S1 Fig — (TIF) [file pone.0251338.s001.tif]

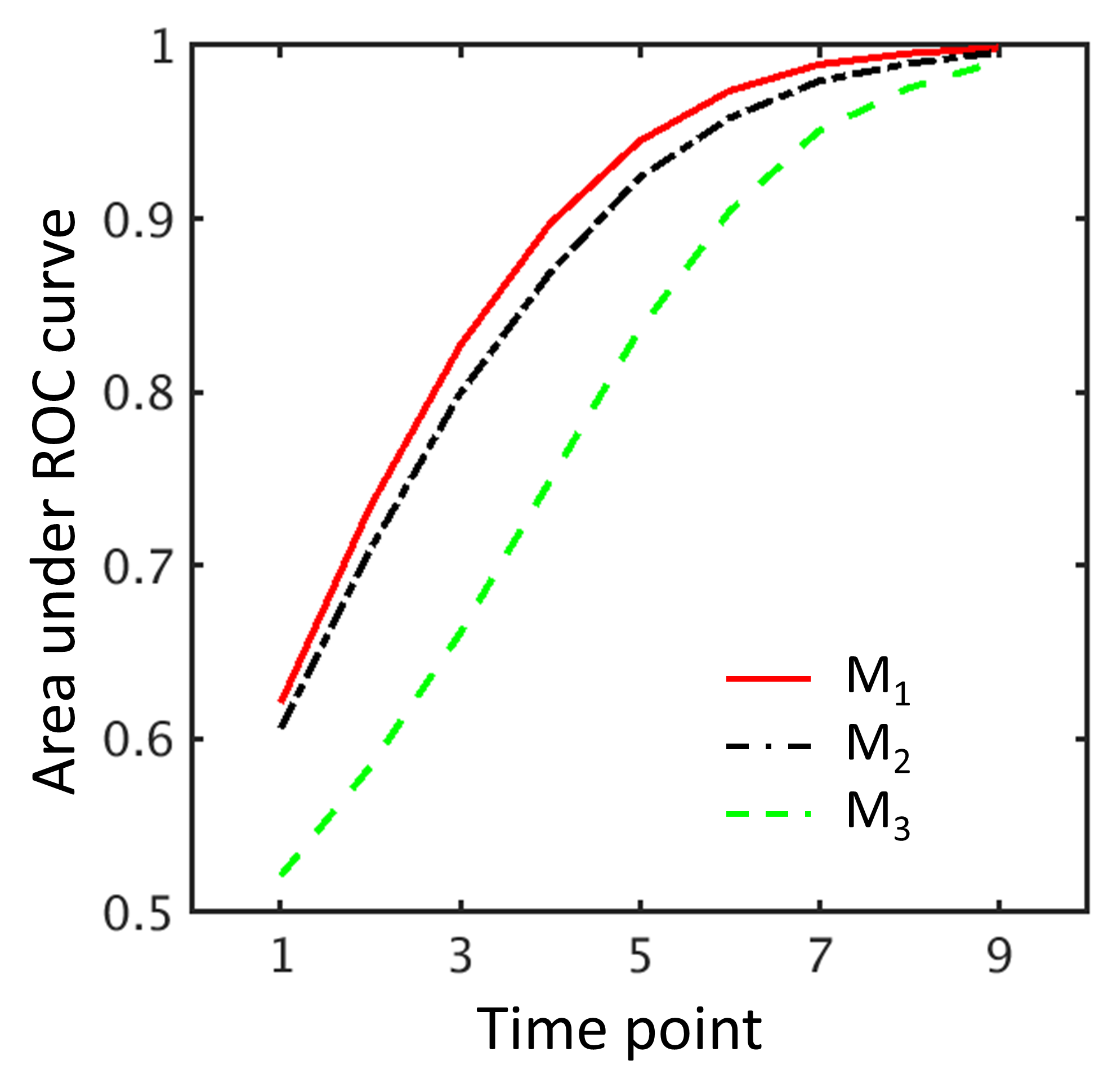

Supplement: S2 Fig — Area under the ROC curve for measures 1–3 (M1, M2, and M3), nine time points. (TIF) [file pone.0251338.s002.tif]

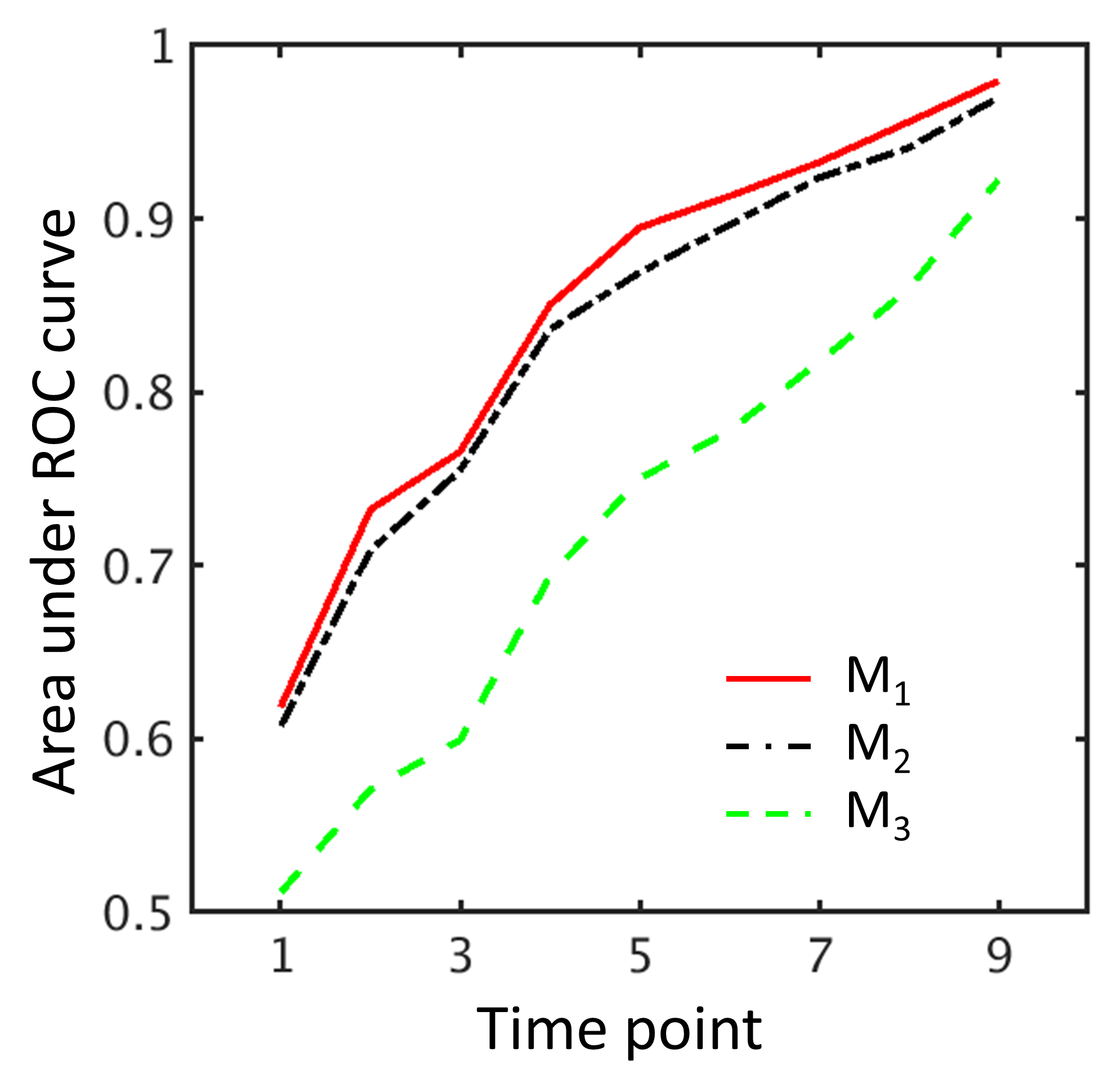

Supplement: S3 Fig — Area under the ROC curve for measures 1–3 (M1, M2, and M3), nine time points. (TIF) [file pone.0251338.s003.tif]
