# Supplementary material for: Evaluation of a connectivity-based imaging metric that reflects functional decline in Multiple Sclerosis
Source: PLoS One. 2021 Jun 8;16(6):e0251338. doi: 10.1371/journal.pone.0251338 (PMC8186801; doi:10.1371/journal.pone.0251338)
Supplement: S1 Text — (DOCX) [file pone.0251338.s004.docx]

Using parameters reported in the literature and findings from our previous work, we investigated methods for combining connectivity data. Below, we describe these analyses and the results of simulated datasets. Our findings determined the composition of the SFCI reported in the main study.

**Combining pathway functional and structural connectivity measures**

We investigated optimal strategies for mathematically combining the pathway dependent functional and structural connectivity measures, Z_motor_ and Z_cog_. There are three independent pathways – the transcallosal motor pathway (SMC) and the right and left hemisphere pathways connecting the antero-mesial temporal lobe and posterior cingulate cortex (PCC-AMTL). We investigated three mathematical choices:

Measure 1: $M_{1}=\frac{1}{3}[Z_{motor}+Z_{cog}^{Left}+Z_{cog}^{Right}]$

Measure 2: $M_{2}=\frac{1}{2}[Z_{motor}+Min\left( Z_{cog}^{Left},Z_{cog}^{Right} \right)]$

Measure 3: $M_{3}=\sqrt{\frac{1}{3}\left[ {(Z_{motor})}^{2}+{(Z_{cog}^{Left})}^{2}+{(Z_{cog}^{Right})}^{2} \right]}$

In order to study the relative sensitivity to change over time of each of these combined measures, we performed simulations of the progression of functional and structural connectivity in the pathways. We then compared the sensitivity to the change in different disease progression scenarios using receiver operating characteristic (ROC) methods [1].

**Simulations**

We ran simulations of pathway disease progression using the following assumptions, supported by evidence from the MS literature: 1. Structural connectivity, for which we are using pathway-averaged radial diffusivity, progresses over time at a rate of 1.7% per year [2]. 2. Functional connectivity and structural connectivity are correlated with r = 0.5 [3]. True and false positive rates for detection of progression were determined by generating null hypothesis (i.e. no progression) and alternate hypothesis distributions (i.e. progression) of structural and functional connectivity change using normally distributed random values and the assumptions above.

Two progression scenarios were investigated:

1. All pathways progress for each time point.
2. Two out of 3 pathways randomly progress for each time point. Each pathway is equally likely to not progress at each time point.

S1 Fig. shows a ROC plot for M_1_ in progression scenario 1. The area under the ROC curve is typically taken as the performance indicator for an ROC analysis. S2 Fig. shows the area under the ROC curve for M_1_, M_2_, and M_3_ for progression scenario 1 across nine time points. S3 Fig. shows the same for progression scenario 2.

In S2 Fig. and S3 Fig., we see that M_1_ and M_2_ have similar performance. Given that, and the fact that M_2_ will be more suitable for a situation where one of the cognitive pathways progresses consistently more than the other, we opted for M_2_ as our metric for the analysis reported in the main study.

**References**

1. Fawcett T. An Introduction to ROC Analysis. Pattern Recognition Letters. 2006;27:861-74. doi: 10.1016/j.patrec.2005.10.010.

2. Harrison DM, Caffo BS, Shiee N, Farrell JA, Bazin PL, Farrell SK, et al. Longitudinal changes in diffusion tensor-based quantitative MRI in multiple sclerosis. Neurology. 2011;76(2):179-86. PubMed PMID: 21220722.

3. Lowe MJ, Koenig KA, Beall EB, Sakaie KA, Stone L, Bermel R, et al. Anatomic connectivity assessed using pathway radial diffusivity is related to functional connectivity in monosynaptic pathways. Brain Connect. 2014;4(7):558-65. doi: 10.1089/brain.2014.0265. PubMed PMID: 25117651.
