# Supplementary material for: Evaluation of a connectivity-based imaging metric that reflects functional decline in Multiple Sclerosis
Source: PLoS One. 2021 Jun 8;16(6):e0251338. doi: 10.1371/journal.pone.0251338 (PMC8186801; doi:10.1371/journal.pone.0251338)
Supplement: Attachment — Submitted filename: Response_to_reviewers.docx [file pone.0251338.s006.docx]

Thank you again to the reviewers for the time and effort applied to our manuscript. Please find our responses to your comments below.

**Reviewer #1:**

1. Although the authors improved readability of the paper a lot, the references are too old. Cognition in MS is highly topic in recent years and I believe there are more updated relevant works in the literature.

Updated references have been added. Please note that using the “track changes” function to note reference changes made the manuscript very difficult to read. Rather, the new references are in red type.

Abstract

2. 3 digits after decimal point is enough for the p value description and 2 digits for r values.

This was modified.

Materials and methods

3. Line 176: Please rephrased as “176 axial slices and 0.94 mm slice thickness”

This was modified for each scan description.

4. “Acquisition time (TA)” would be better instead of “Scan length”

This was modified for each scan description.

5. Please provide abbreviations such as inversion time (TI), TE, TR ….

These definitions were added under Scan 1.

Results

6. In general each table and figure caption should include the abbreviations.

Abbreviations have been added to the figures and tables. For the sake of space, a number of captions refer the reader to abbreviations under Table 3.

7. Figure 4: Please provide all abbreviations MS, SMC… and descriptions like (A) shows sensorimotor cortex etc. in the figure caption.

Abbreviations and additional description have been added for this figure.

**Reviewer #2:**

1. The only concern of mine remaining is that full data availability has not been definitely addressed.

As previously mentioned, the Cleveland Clinic has strict requirements for data sharing, including review by the Institutional Review Board, legal department, and cybersecurity. We hoped to receive full approval by the resubmission deadline, but still await approval from the Cleveland Clinic legal department. We expect full approval by the end of April 2021. We assure the reviewers and editors that we have moved as quickly as possible to gain approval, and apologize for the delay. The data supporting this work is uploaded to Figshare. As soon as approval is received, the data will be published publically and we will notify the Editor. In the meantime, we have made a private link available to the reviewers and editors:

<https://figshare.com/s/db2f3272926c015412fb>
